# The first complete mitochondrial genome of the Mariana Trench *Freyastera benthophila* (Asteroidea: Brisingida: Brisingidae) allows insights into the deep‐sea adaptive evolution of Brisingida

**DOI:** 10.1002/ece3.4427

**Published:** 2018-10-31

**Authors:** Wendan Mu, Jun Liu, Haibin Zhang

**Affiliations:** ^1^ Institute of Deep‐Sea Science and Engineering Chinese Academy of Sciences Sanya China; ^2^ University of Chinese Academy of Sciences Beijing China

**Keywords:** adaptive evolution, Brisingida, deep sea, *Freyastera benthophila*, mitochondrial genome

## Abstract

Starfish (phylum Echinodermata) are ecologically important and diverse members of marine ecosystems in all of the world's oceans, from the shallow water to the hadal zone. The deep sea is recognized as an extremely harsh environment on earth. In this study, we present the mitochondrial genome sequence of Mariana Trench starfish *Freyastera benthophila*, and this study is the first to explore in detail the mitochondrial genome of a deep‐sea member of the order Brisingida. Similar to other starfish, it contained 13 protein‐coding genes, two ribosomal RNA genes, and 22 transfer RNA genes (duplication of two tRNAs: *trnL* and *trnS*). Twenty‐two of these genes are encoded on the positive strand, while the other 15 are encoded on the negative strand. The gene arrangement was identical to those of sequenced starfish. Phylogenetic analysis showed the deep‐sea Brisingida as a sister taxon to the traditional members of the Asteriidae. Positive selection analysis indicated that five residues (8 N and 16 I in *atp8*, 47 D and 196 V in *nad2*, 599 N in *nad5*) were positively selected sites with high posterior probabilities. Compared these features with shallow sea starfish, we predict that variation specifically in *atp8*,* nad2*, and *nad5* may play an important role in *F. benthophila*'s adaptation to deep‐sea environment.

## 1 INTRODUCTION

The class Asteroidea (sea stars and starfish) is one of the most familiar and diverse groups of the phylum Echinodermata with a long paleontological history, including nearly 1,800 species grouped into 35 families (Clark & Downey, 1992; Matsubara, Komatsu, & Wada, 2004). They are present in all of the world's oceans and occur from intertidal to abyssal, and they are most diverse in the Indo‐Pacific and tropical Atlantic regions (Mah & Blake, 2012). To date, the phylogenetic relationships of these starfish have not yet been fully resolved (Knott & Wray, 2000).

Because of its maternal inheritance, and low frequency of gene recombination, mitochondrial genes (e.g., COI) are widely used for phylogenetic analysis (Boore, 1999). Compared to one gene, complete mitogenomes include more genetic information and usually could obtain more accurate phylogenetic relationship and therefore have become more popular in recent years (Fan, Hu, Wen, & Zhang, 2011; Shen, Ma, Ren, & Zhao, 2009; Shen et al., 2017). Up to now, the complete mitochondrial genomes have been reported in many marine organisms, such as sea cucumber (Fan et al., 2011; Perseke et al., 2010; Scouras, Beckenbach, Arndt, & Smith, 2004; Sun, Qi, & Kong, 2010), sea urchin (Cantatore, Roberti, Rainaldi, Gadaleta, & Saccone, 1989; De Giorgi, Martiradonna, Lanave, & Saccone, 1996; Qureshi & Jacobs, 1993), brittle star (Perseke et al., 2008, 2010; Scouras et al., 2004; Smith, Arndt, Gorski, & Fajber, 1993), sea lily (Perseke et al., 2008; Scouras & Smith, 2006), shellfish (Plazzi, Ribani, & Passamonti, 2013; Ren, Liu, Jiang, Guo, & Liu, 2010), and crab (Liu & Cui, 2010; Yang & Yang, 2008).

Animal mitogenome is typically always circular molecule, except for some classes of cnidarians (Bridge, Cunningham, Schierwater, Desalle, & Buss, 1992). It contains 37 genes in general: 13 protein‐coding genes (PCGs) (cytochrome *c* oxidase subunits I–III [*cox1*‐*cox3*], NADH dehydrogenase subunits 1–6 and 4L [*nad1*‐*6*,* nad4L*], ATP synthase subunits 6 and 8 [*atp6*,* atp8*], apocytochrome *b* [*cob*]), two ribosomal RNAs (*12S* and *16S*), and 22 transfer RNAs (tRNAs). All 13 protein‐coding genes play key roles in oxygen usage and energy metabolism (Boore, 1999; Xu et al., 2007). Because variation in mitochondrial protein‐coding genes that involved in oxidative phosphorylation can directly influence metabolic performance, an increasing number of researches related to adaptive evolution of these genes have been reported (Maliarchuk, 2011; Xu et al., 2007; Yu, Wang, Ting, & Zhang, 2011; Zhou, Shen, Irwin, Shen, & Zhang, 2014). Despite strong functional constraints, mitochondrial DNA may be subject to positive directional selection in response to pressures from extreme harsh environment (Tomasco & Lessa, 2011). Indeed, mtDNA analyses have demonstrated the existence of adaptive evolution in the ATP synthase genes of the sea anemones, alvinocaridid shrimp, and galliform birds (Sun, Hui, Wang, & Sha, 2018; Zhang, Zhang, Wang, Zhang, & Lin, 2017; Zhou et al., 2014); the NADH dehydrogenase genes of sea anemones, alvinocaridid shrimp, Tibetan horses, and Chinese snub‐nosed monkeys (Sun et al., 2018; Xu et al., 2007; Yu et al., 2011; Zhang et al., 2017); the cytochrome *b* gene of cetaceans and alpacas (da Fonseca, Johnson, O'Brien, Ramos, & Antunes, 2008); and the cytochrome *c* oxidase genes of Tibetan antelope and anthropoids (Adkins & Honeycutt, 1994; Luo et al., 2008).

Here, we report the complete mitogenome of the starfish *Freyastera benthophila*, which collected from Mariana Trench at 5,463 m depth. *Freyastera benthophila* exist in abyssal and is mainly distributed in the southern Pacific, eastern Pacific off California, mid‐Atlantic (between Azores and Spain), the Bay of Bengal, and Biscay, ranging from 4,250 to 5,000 m depth (Downey, 1986). The mitogenome features, organization, codon usage, and gene arrangement information were presented. The phylogenetic relationships between *F. benthophila* and 19 other species from Echinodermata were analyzed. To infer the deep‐sea adaptive evolution, positive selection analysis of mitochondrial genes was also performed.

## 2 MATERIALS AND METHODS

### 2.1 Sample collection and DNA extraction

The specimen was collected at Mariana Trench, in June 2016 (10°51.0971′N, 141°57.2705′E, at 5,463 m depth). The collection was accomplished by deep‐sea human occupied vehicle (HOV) “Jiao Long” during an expedition. The specimen was preserved in 95% ethanol. Total genomic DNA was extracted from ethanol‐fixed tissue with tissue DNA kit (Omega Bio‐Tek) and stored at −20°C.

### 2.2 PCR amplification and DNA sequencing

The universal metazoan primers for mtDNA were used in PCR. Three fragments *cox3*,* cob*, and *16S* were successfully amplified with the primers cox3F + cox3R (Boore, Macey, & Medina, 2005), cobF424 + cobR876 (Boore & Brown, 2000), and 16SarL + 16SbrH (Boore et al., 2005). In addition, partial sequences of *cox1*,* cox2*,* nad4L*,* atp6*,* nad4*, and *nad5* were amplified with the degenerate primers from conserved regions of other starfish in GenBank. The remaining gaps were amplified with the species‐specific primers designed according to the obtained sequences. Finally, the whole mitogenome was amplified based on eight pairs of primers (Table 1).

**Table 1:** Primers used for amplifying and sequencing the mitogenome of *Freyastera benthophila*

Name	Sequence (5′–3′)	Region	Location
W1‐F	CCGCAAGAGTCGAAAGAG	*cox1*	3473–3490
W1‐R	TCAAGGAGTCGTGGCATT	*cox2*	5448–5465
W2‐F	TAGCTCTTTCCCGAACAC	*nad4L*	4993–5010
W2‐R	GAACCGTAAACACTATCTGCT	*cox3*	7233–7253
W3‐F	GACTCGCAGCTAATCTTACA	*atp6*	6466–6485
W3‐R	CAAGACCGTATCCACCTAAC	*nad4*	8630–8649
W4‐F	CCCTCCTTCCAACCCTCATC	*nad4*	8287–8306
W4‐R	CACCCATCTTTCGTAGGTCTTGT	*nad5*	10735–10757
W5‐F	CCACCGCTACTTCTCAACAT	*nad5*	10545–10564
W5‐R	TAGAGCGAAGGATTGCATAG	*cob*	12860–12879
W6‐F	CCACCTATTCTTCCTTCACC	*cob*	12614–12633
W6‐R	GCATAATCATTTGCCTCTTA	*16S*	15183–15202
W7‐F	AGCTCGATAGGGTCTTCTCGTC	*16S*	15063–15084
W7‐R	GCAGTGGCATTGTTGACTTTGA	*nad1*	2040–2061
W8‐F	AGCTAACGGCTGAAACAATC	*nad1*	1957–1976
W8‐R	TTCCTGCGTAATGGGCTA	*cox1*	3902–3919

PCRs were performed with a gradient machine (Applied Biosystems Inc.). The cycling was set up with an initial denature step at 94°C for 5 min, followed by 35 cycles (94°C for 30 s, 45–55°C for 1 min, 72°C 1–3 min), and a final extension was executed at 72°C for 10 min. LA‐PCR was carried out in a 20 μl reaction volume containing 12.6 μl ddH_2_O, 2 μl 10 × LA‐PCR buffer (Mg^2+^ plus, Takara), 3.2 μl dNTP mix (2.5 mM each), 0.5 μl each primer (10 μM), 0.2 μl LA Taq DNA polymerase (5 U/μl, Takara), and 1 μl DNA template (50 ng/μl). PCR products were electrophoresed on a 1.0% agarose gel and purified with gel extraction kit (Omega Bio‐Tek) and sequenced with ABI 3730x1 DNA analyzer (Applied Biosystems Inc.).

### 2.3 Gene annotation and Sequence analysis

Raw sequencing reads were first processed using Phred with the quality score 20 and assembled in Phrap with default parameters (Ewing & Green, 1998; Ewing, Hillier, Wendl, & Green, 1998). Then, all assemblies and sequence quality were verified manually in Consed (Gordon, Abajian, & Green, 1998). DOGMA (Wyman, Jansen, & Boore, 2004), ORFfinder (http://www.ncbi.nlm.nih.gov/projects/gorf/orfig.cgi), and BLAST (http://blast.ncbi.nlm.nih.gov/Blast.cgi) were used to identify protein‐encoding genes and rRNA genes. The tRNA genes were identified by tRNAscan‐SE 1.21 (Lowe & Eddy, 1997) and ARWEN 1.2.3.c (Laslett & Canbäck, 2008). Secondary structures for tRNAs were drawn using MITOS Web server (Bernt et al., 2013). Codon usage analysis was estimated with CodonW 1.4.4 (Peden, 1999). The mitochondrial gene map was drawn with GenomeVx (Conant & Wolfe, 2008).

### 2.4 Phylogenetic analysis

Twenty echinoderm mt genomes including the one obtained in this study were used for phylogenetic analysis. All complete mtDNA sequences vailable in GenBank are listed in Table 2. Crinoidea is generally considered as the earliest diverged group of echinoderms (Scouras & Smith, 2006). In this study, *Antedon mediterranea* (Crinoidea) was rooted as the out‐group. The amino acid sequence from each of 13 protein‐coding genes was aligned separately using Clustal ×2.0 (Larkin et al., 2007), and then, the relatively poor homologous sequence was eliminated. The aligned amino acid sequences were concatenated into a single dataset. The phylogenetic reconstruction approach was performed using neighbor joining (NJ) and maximum likelihood (ML) with MEGA 5.0 (Tamura et al., 2011). The assessment of node reliability was performed using 1,000 bootstrap replicates.

**Table 2:** List of taxa used in the phylogenetic analysis

Taxon	Classification	Accession number	References
Crinoidea			
* Antedom mediterranea*	Crinoidea; Articulata; Comatulida; Antedonidae	NC_010692	Perseke et al. (2008)
Ophiuroidea			
* Ophiocomina nigra*	Ophiuroidea; Ophiuridea; Ophiurida; Ophiurina; Gnathophiurina; Ophiocomidae	NC_013874	Perseke et al. (2010)
* Astrospartus mediterraneus*	Ophiuroidea; Ophiuridea; Euryalida; Gorgonocephalidae	NC_013878	Perseke et al. (2010)
Echinoidea			
* Strongylocentrotus purpuratus*	Echinoidea; Euechinoidea; Echinacea; Echinoida; Strongylocentrotidae	NC_001453	Qureshi and Jacobs (1993)
* Echinocardium cordatum*	Echinoidea; Euechinoidea; Atelostomata; Spatangoida; Loveniidae	NC_013881	Perseke et al. (Unpublished)
* Paracentrotus lividus*	Echinoidea; Euechinoidea; Echinacea; Echinoida; Echinidae	NC_001572	Cantatore et al. (1989)
Holothuroidea			
* Cucumaria miniata*	Holothuroidea; Dendrochirotacea; Dendrochirotida; Cucumariidae	NC_005929	Scouras et al. (2004)
* Apostichopus japonicus*	Holothuroidea; Aspidochirotacea; Aspidochirotida; Stichopodidae	NC_012616	Sun et al. (2010)
* Holothuria forskali*	Holothuroidea; Aspidochirotacea; Aspidochirotida; Holothuriidae	NC_013884	Perseke et al. (2010)
* Parastichopus nigripunctatus*	Holothuroidea; Aspidochirotacea; Aspidochirotida; Stichopodidae	NC_013432	Sasaki and Hamaguchi (Unpublished)
* Stichopus horrens*	Holothuroidea; Aspidochirotacea; Aspidochirotida; Stichopodidae	NC_014454	Fan et al. (2011)
Asteroidea			
* Freyastera benthophila*	Asteroidea; Forcipulatacea; Brisingida; Brisingidae	MG563681	This study
* Aphelasterias japonica*	Asteroidea; Forcipulatacea; Forcipulatida; Asteriidae	NC_025766	Tang et al. (2014)
* Pisaster ochraceus*	Asteroidea; Forcipulatacea; Forcipulatida; Asteriidae	X55514	Smith, Banfield, Doteval, Gorski, and Kowbel (1990)
* Asterias amurensis*	Asteroidea; Forcipulatacea; Forcipulatida; Asteriidae	NC_006665	Matsubara et al. (2005)
* Astropecten polyacanthus*	Asteroidea; Valvatacea; Paxillosida; Astropectinidae	NC_006666	Matsubara et al. (2005)
* Luidia quinaria*	Asteroidea; Valvatacea; Paxillosida; Luidiidae	NC_006664	Matsubara et al. (2005)
* Acanthaster brevispinus*	Asteroidea; Valvatacea; Valvatida; Acanthasteridae	NC_007789	Yasuda et al. (2006)
* Acanthaster planci*	Asteroidea; Valvatacea; Valvatida; Acanthasteridae	NC_007788	Yasuda et al. (2006)
* Patiria pectinifera*	Asteroidea; Valvatacea; Valvatida; Asterinidae	NC_001627	Asakawa et al. (1995)

### 2.5 Positive selection analysis

To evaluate the variation in selective pressure between deep‐sea *F. benthophila* and other eight shallow sea starfish, we used a codon‐based likelihood approach implemented in the CODEML program of the pamlX package (Xu & Yang, 2013; Yang, 2007). All models correct the transition/transversion rate and codon usage biases (F3 × 4). The branch model tests were used to analyze the difference of selective pressure between the deep‐sea and shallow sea starfish. The “one‐ratio” model (model 0), “free‐ratio” model (model 1), and “two‐ratio” model were used in the combined dataset of 13 protein‐coding genes. Considering that positive selection may occur in some amino acids during the evolution of a protein, we used two branch site models (A and A null). Bayes empirical Bayes (Yang, Wong, & Nielsen, 2005) analysis was used to calculate the posterior probabilities of a specific codon site.

## 3 RESULTS AND DISCUSSION

### 3.1 General features

**Figure 1:**
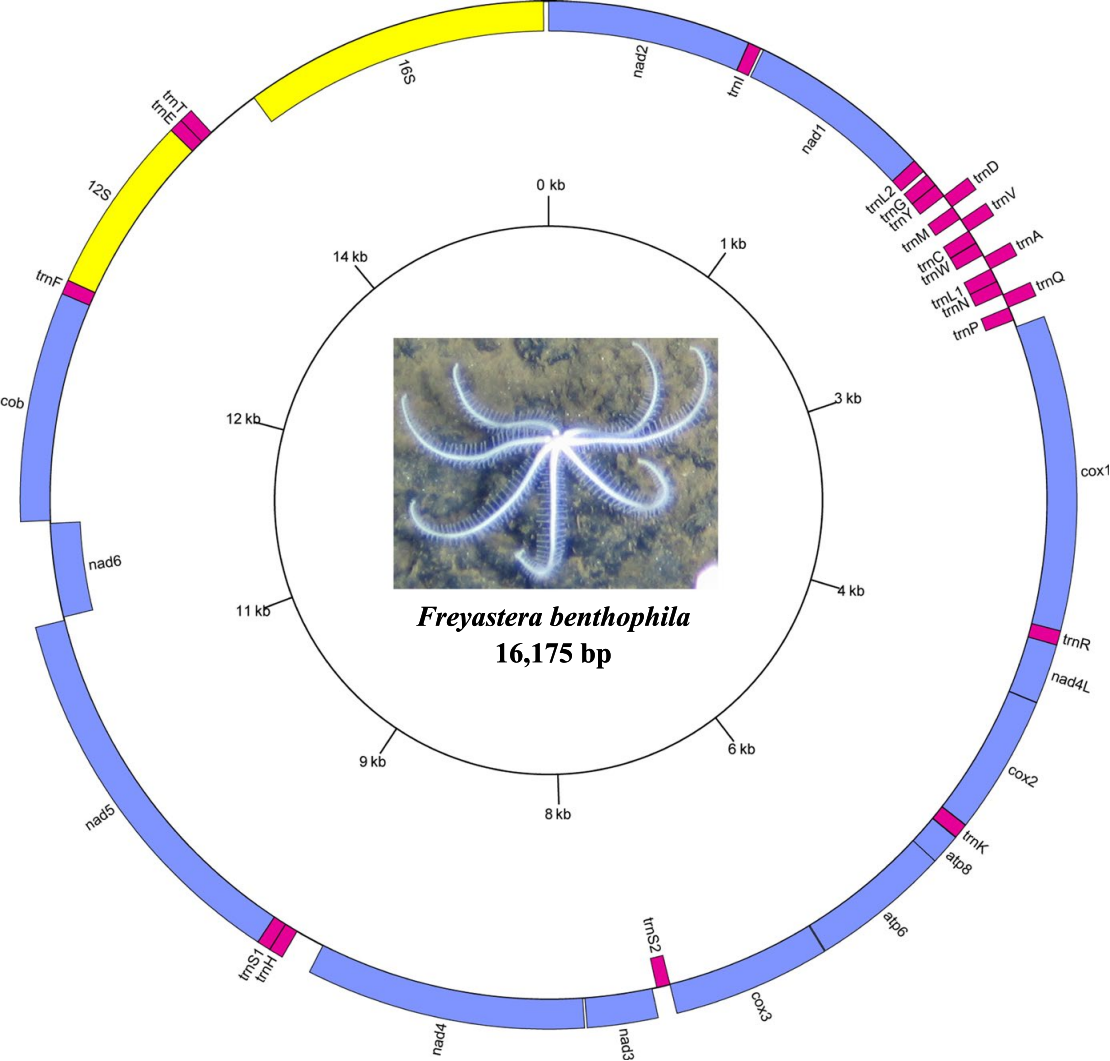
Mitochondrial gene map of *Freyastera benthophila*. All of 37 genes are encoded on the both strands. Genes for proteins and rRNAs are shown with standard abbreviation. Genes for tRNAs are designated by a single letter for the corresponding amino acid with two leucine tRNAs and two serine tRNAs differentiated by numerals

**Figure 2:**
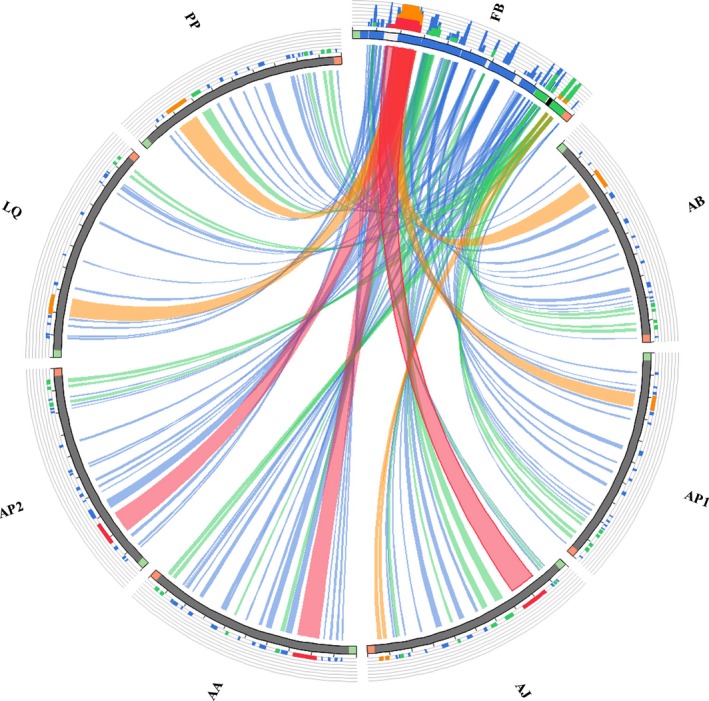
The synteny and identity level of *Freyastera benthophila* mitogenome against each of the other seven starfish mitogenomes. Ideograms and ribbons represent the similarity pairwise blastn searches. In *F. benthophila* ideogram, the 13 coding genes are colored in blue, control regions are colored in black, and rRNAs are colored in green. The figure was produced using Circoletto (Darzentas, 2010). FB (*F. benthophila*), AB (*Acanthaster brevispinus*), AP1 (*Acanthaster planci*), AJ (*Aphelasterias japonica*), AA (*Asterias amurensis*), AP2 (*Astropecten polyacanthus*), LQ (*Luidia quinari*a), PP (*Patiria pectinifera*)

The mitogenome of the *F. benthophila* is a 16,175‐bp circular molecule (Figure 1) with a nucleotide composition of 34.70% A, 21.13% C, 10.65% G, and 33.53% T bases. The genome has an overall A + T content of 68.23%, which appears to be high for Asteroidea. Among the eight species in Asteroidea, the lowest A + T content is 56.34% in *Acanthaster planci* (Table 3). *Freyastera benthophila* has the smallest complete mitogenome found in Asteroidea thus far. The size of Asteroidea mitogenomes ranged from 16,524 bp in *Luidia quinaria* to 16,175 bp in *F. benthophila* (Table 3). The synteny and identity level between *F. benthophila* and each of the other seven starfish mitogenomes is shown in Figure 2. The lack of similarity between *F. benthophila* and *L. quinaria* is the most obvious feature in the plot.

**Table 3:** Genomic characteristics of Asteroidea mtDNAs

	*Freyastera benthophila*	*Asterias amurensis*	*Astropecten polyacanthus*	*Luidia quinaria*	*Aphelasterias japonica*	*Acanthaster brevispinus*	*Acanthaster planci*	*Patiria pectinifera*
Entire genome length (bp)	16,175	16,427	16,304	16,524	16,215	16,254	16,234	16,260
Entire genome A + T%	68.23	65.45	64.00	62.98	64.32	56.37	56.34	61.27
Protein‐coding gene length (bp)	11,506	11,488	11,539	11,506	11,504	11,488	11,491	11,501
Protein‐coding gene A + T%	67.15	64.45	62.48	61.02	62.95	55.95	55.64	60.12
*12S* gene length (bp)	891	893	901	884	900	928	929	897
*12S* gene A + T%	65.66	61.70	62.04	59.16	62.22	53.45	54.04	58.86
*16S* gene length (bp)	1,602	1,620	1,629	1,751	1,602	1,545	1,549	1,531
*16S* gene A + T%	72.28	70.19	69.12	69.67	69.66	55.34	56.04	66.49
tRNA length (bp)	1,557	1,580	1,561	1,563	1,566	1,546	1,550	1,585
tRNA A + T%	60.65	67.22	67.01	66.54	66.99	61.45	61.35	64.35
Largest NCR length (bp)	284	483	395	402	281	551	531	445
Largest NCR A + T%	67.25	63.98	64.56	70.15	62.63	53.90	54.99	59.78
*nad2*	1062 (ATG/TAG)	1062 (GTG/TAA)	1068 (GTG/TAA)	1068 (GTG/TAA)	1062 (ATG/TAG)	1065 (ATG/TAG)	1065 (ATG/TAG)	1065 (ATG/TAA)
*nad1*	972 (ATG/TAG)	978 (GTG/TAA)	976 (ATG/T‐)	978 (GTG/TAG)	978 (GTG/TAA)	981 (GTG/TAG)	981 (GTG/TAG)	981 (GTG/TAG)
*cox1*	1557 (ATG/TAA)	1551 (ATG/TAA)	1554 (ATG/TAA)	1554 (ATG/TAA)	1552 (ATG/T‐)	1553 (ATG/TA‐)	1553 (ATG/TA‐)	1554 (ATG/TAA)
*nad4L*	297 (ATG/TAA)	288 (ATG/TAA)	297 (ATT/TAA)	297 (ATT/TAA)	297 (ATC/TAA)	297 (ATT/TAA)	297 (ATT/TAA)	297 (ATT/TAA)
*cox2*	690 (ATG/TAA)	690 (ATG/TAA)	688 (ATG/T‐)	693 (ATG/TAG)	690 (ATG/TAA)	688 (ATG/T‐)	688 (ATG/T‐)	688 (ATG/T‐)
*atp8*	168 (ATG/TAA)	168 (ATG/TAA)	168 (ATG/TAA)	168 (ATG/TAA)	168 (ATG/TAA)	165 (ATG/TAA)	165 (ATG/TAA)	165 (ATG/TAA)
*atp6*	693 (ATG/TAA)	693 (ATG/TAA)	693 (ATG/TAA)	693 (ATG/TAA)	693 (ATG/TAA)	693 (ATG/TAA)	693 (ATG/TAA)	693 (ATG/TAA)
*cox3*	783 (TAG/TAA)	780 (ATG/TAA)	783 (ATG/TAA)	783 (ATG/TAA)	783 (ATG/TAA)	783 (ATG/TAA)	783 (ATG/TAA)	783 (ATG/TAA)
*nad3*	351 (ATG/TAA)	351 (ATG/TAA)	351 (ATT/TAA)	351 (ATT/TAG)	351 (ATG/TAA)	351 (ATT/TAA)	351 (ATT/TAA)	333 (ATT/TAG)
*nad4*	1386 (ATG/TAA)	1383 (ATG/TAA)	1380 (ATG/TAA)	1380 (ATG/TAA)	1383 (ATG/TAA)	1383 (ATG/TAG)	1383 (ATG/TAA)	1383 (ATG/TAA)
*nad5*	1920 (ATG/TAA)	1917 (ATG/TAA)	1905 (ATG/TAA)	1911 (ATG/TAA)	1920 (ATG/TAA)	1902 (ATG/TAA)	1902 (ATG/TAA)	1932 (GTG/TAA)
*nad6*	489 (ATG/TAG)	489 (ATG/TAG)	492 (ATG/TAA)	492 (ATG/TAA)	489 (ATG/TAG)	489 (ATG/TAG)	489 (ATG/TAG)	489 (ATG/TAA)
*cob*	1138 (ATG/T‐)	1138 (ATG/T‐)	1138 (ATG/T‐)	1138 (ATG/T‐)	1138 (ATG/T‐)	1138 (ATG/T‐)	1138 (ATG/T‐)	1138 (ATG/T‐)
Reference	This study	Matsubara et al. (2005)	Matsubara et al. (2005)	Matsubara et al. (2005)	Tang et al. (2014)	Yasuda et al. (2006)	Yasuda et al. (2006)	Asakawa et al. (1995)

The genome encodes 37 genes including 13 protein‐coding genes (PCGs), two rRNA genes, and 22 tRNA genes (duplication of two tRNAs: *trnL* and *trnS*) on both strands. Fifteen of the genes are encoded on the negative strand, while the other 22 are encoded on the positive strand. A total of 22 noncoding regions were found, with the largest continuous region (284 bp, A + T = 67.25%) located between *trnT* and *16S*. Due to its AT richness, we predict that this part is mitochondrial control region. Furthermore, we found four overlaps: *trnC*/*trnV*,* trnA*/*trnL*_*1*_, *atp8*/*atp6*, and *cox3*/*trnS*_*2*_. Table 4 presents a summary of the organization of *F. benthophila* mitogenome. The complete mitochondrial DNA sequence has been deposited in GenBank (Accession Number: MG563681).

**Table 4:** Gene content of the *Freyastera benthophila* mitogenome

Gene	Location		Size		Codon		Intergenic nucleotide (bp)	Strand
	Start	End	Nucleotide (bp)	Amino acid	Start	Stop		
*nad2*	1	1062	1,062	353	ATG	TAG	0	L
*trnI*	1064	1132	69				1	L
*nad1*	1147	2118	972	323	ATG	TAG	14	L
*trnL*_*2*_	2119	2191	73				0	L
*trnG*	2216	2284	69				24	L
*trnY*	2286	2356	71				1	L
*trnD*	2357	2426	70				0	H
*trnM*	2428	2500	73				1	L
*trnV*	2509	2579	71				8	H
*trnC*	2578	2648	71				−2	L
*trnW*	2651	2721	71				2	L
*trnA*	2735	2804	70				13	H
*trnL*_*1*_	2804	2875	72				−1	L
*trnN*	2876	2947	72				0	L
*trnQ*	2951	3022	72				3	H
*trnP*	3023	3093	71				0	L
*cox1*	3130	4686	1,557	518	ATG	TAA	36	H
*trnR*	4687	4757	71				0	H
*nad4L*	4758	5054	297	98	ATG	TAA	0	H
*cox2*	5056	5745	690	229	ATG	TAA	1	H
*trnK*	5747	5821	75				1	H
*atp8*	5824	5991	168	55	ATG	TAA	2	H
*atp6*	5976	6668	693	230	ATG	TAA	−16	H
*cox3*	6673	7455	783	260	ATG	TAA	4	H
*trnS*_*2*_	7454	7524	71				−2	L
*nad3*	7549	7899	351	116	ATG	TAA	24	H
*nad4*	7911	9296	1,386	461	ATG	TAA	11	H
*trnH*	9448	9517	70				151	H
*trnS*_*1*_	9519	9586	68				1	H
*nad5*	9587	11506	1,920	639	ATG	TAA	0	H
*nad6*	11524	12012	489	162	ATG	TAG	17	L
*cob*	12027	13164	1,138	379	ATG	T‐	14	H
*trnF*	13165	13235	71				0	H
*12S*	13236	14126	891				0	H
*trnE*	14127	14194	68				0	H
*trnT*	14195	14263	69				0	H
*16S*	14548	16149	1,602				284	L

### 3.2 Protein‐coding genes

With regard to PCGs, nine (*cox1*‐*cox3*,* nad3*‐*nad5*,* nad4L*,* cob*,* atp6*, and *atp8*) are encoded by the positive strand, and the remaining three (*nad1*,* nad2*, and *nad6*) are encoded by the negative strand. These features have been observed in all Asteroidea mitogenomes published so far. Thirteen PCGs initiate with the standard start codon ATG. Most of PCGs terminate with the stop codon TAA (9 of 13), and three genes terminate with the stop codon TAG. Incomplete termination codon T is used by *cob*. However, mitogenomes often use a variety of nonstandard initiation codons (Wolstenholme, 1992). Nonstandard initiation codon GTG and incomplete termination codon TA are also used in other starfish (Table 3). The lengths of PCGs are 11,506 bp, and the A + T content is 67.15% higher than that of other Asteroidea species (Table 3).

**Figure 3:**
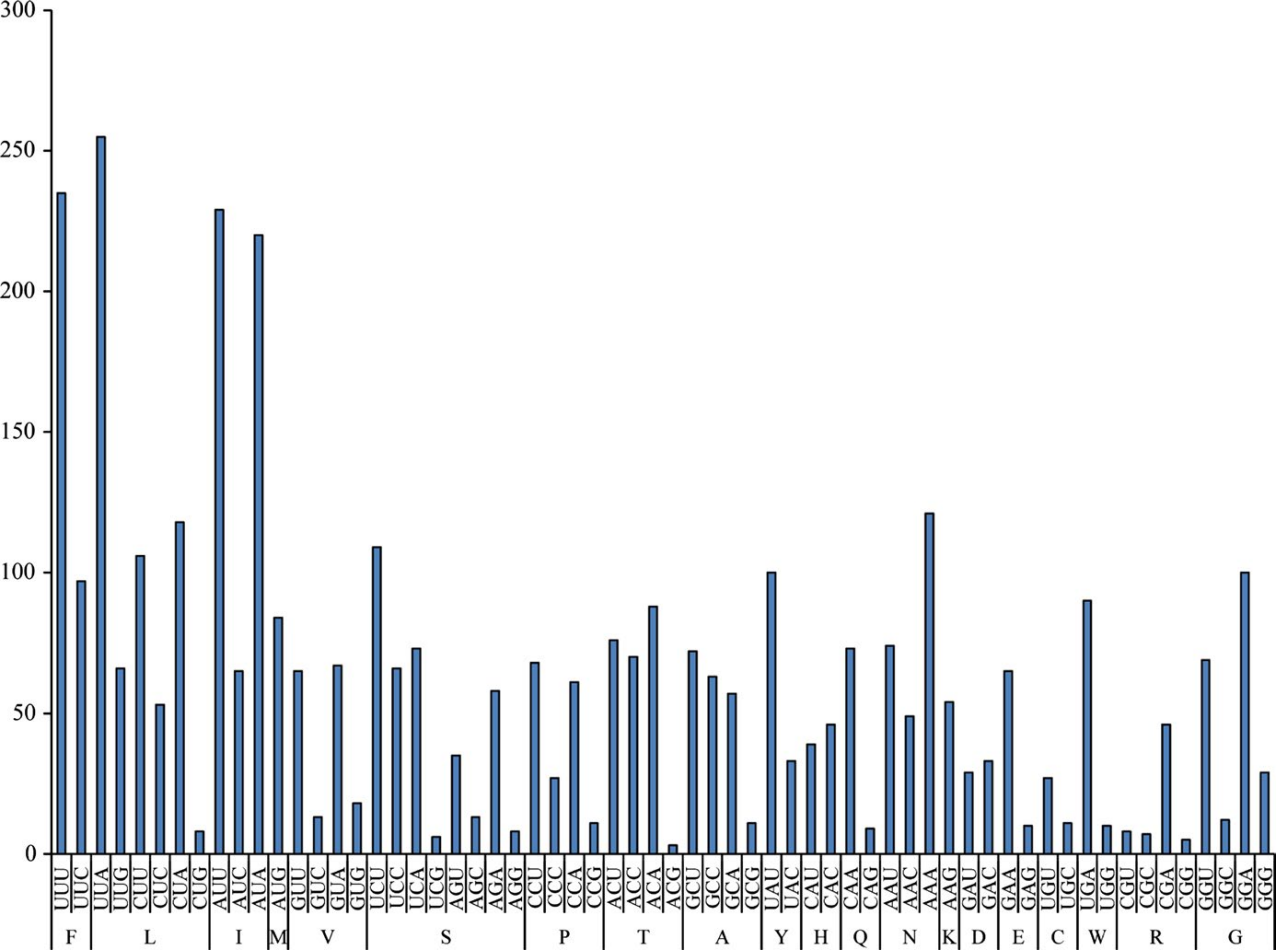
Codon usage in *Freyastera benthophila*. All codons for amino acids have been classified. Each amino acid is designated by a single letter for the corresponding codon. *x*‐axis and *y*‐axis represent the used times of each codon

The codon usage of *F. benthophila* is shown in Figure 3. Among PCGs, leucine (15.85%) and cysteine (0.99%) are the most and the least frequently used amino acids, respectively. Codons, UUA (leucine 6.67%) and ACG (threonine 0.08%), are the most and the least frequently used, respectively. We predict that the richness of A and T occurrence frequency of the mitogenome caused the corresponding amino acid bias to some extent. It is obvious that the A + T content of the third codon position (74.10%) is higher than that of the first (63.43%) and second positions (63.67%).

### 3.3 Ribosomal RNA and transfer RNA genes

Boundaries of both the small and the large ribosomal genes were determined by BLAST and DOGMA. The *16S* and *12S* genes of *F. benthophila* are 1,602 bp (A + T = 72.28%) and 891 bp (A + T = 65.66%) in length, respectively. These lengths are typical for Asteroidea, whereas the AT contents are higher than those of other starfish (Table 3).

We analyzed the entire mitogenome sequence of *F. benthophila* and successfully identified 22 tRNA genes based on their potential secondary structures using the tRNAscan‐SE, ARWEN, and MITOS Web server (Table 4, Supporting Information Figure S1). The length of these tRNA genes ranged from 68 bp (*trnS*_*1*_ and *trnE*) to 75 bp (*trnK*). Twenty‐one of these genes displayed a common cloverleaf secondary structure, and the remaining one lacked a DHU arm from *trnS*_*1*_. The D‐stem absence has been found in many other starfish, such as *Acanthaster brevispinus*,* Acanthaster planci*,* Aphelasterias japonica*,* Asterias amurensis*,* L. quinaria*, and *Patiria pectinifera* (Asakawa, Himeno, Miura, & Watanabe, 1995; Matsubara et al., 2005; Tang et al., 2014; Yasuda et al., 2006).

### 3.4 Gene arrangement

Mitochondrial gene arrangement has been demonstrated to be an effective means to solve the deep phylogenetic studies (Boore, 1999; Boore & Brown, 1998). In recent years, some research on mt gene arrangement of echinoderms has been reported (Arndt & Smith, 1998; Perseke et al., 2008, 2010; Scouras et al., 2004).

**Figure 4:**
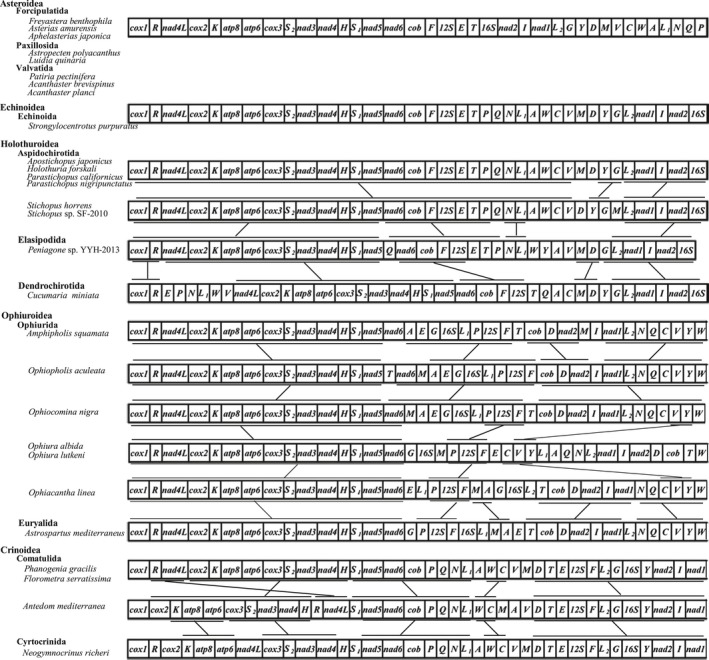
Comparison of mitochondrial gene arrangement in Echinodermata. The bars show identical gene blocks. The noncoding regions are not presented, and gene segments are not drawn to scale

In this study, mitochondrial gene order of echinoderm was compared among species within classes Asteroidea, Echinoidea, Holothuroidea, Ophiuroidea, and Crinoidea (Figure 4). We expected that the mt gene order of starfish may reveal some phylogenetically information. However, the gene component and gene order of eight species of Asteroidea are completely identical to each other. This phenomenon also happened in the class Echinoidea. We obtained 27 complete mt genomes of Echinoidea from NCBI genebank, and the gene component and gene order of 27 species of Echinoidea are also completely identical to each other. Because *Strongylocentrotus purpuratus* has been considered as a model for developmental and systems biology, we took *S. purpuratus* as a representative for Echinoidea in Figure 4 (Sodergren et al., 2006). However, mitochondrial gene order has undergone significant changes in the classes of Holothuroidea, Ophiuroidea, and Crinoidea. Scouras et al. (2004) suggested that it is difficult to resolve the echinoderm phylogeny using the mitochondrial gene rearrangement.

It is interesting that mt gene order of the species in the classes of Asteroidea and Echinoidea is completely identical to each other. If the tRNA is not considered, gene order of PCGs in species within the class Holothuroidea is also the same. This raises the questions: As these species are distributed throughout the world's oceans, why had the mt gene order not been changed and how do they evolve over time. More studies of mt genome species are needed to further investigate whether this pattern is common among starfish, sea urchins, and sea cucumbers.

### 3.5 Phylogenetic analysis

**Figure 5:**
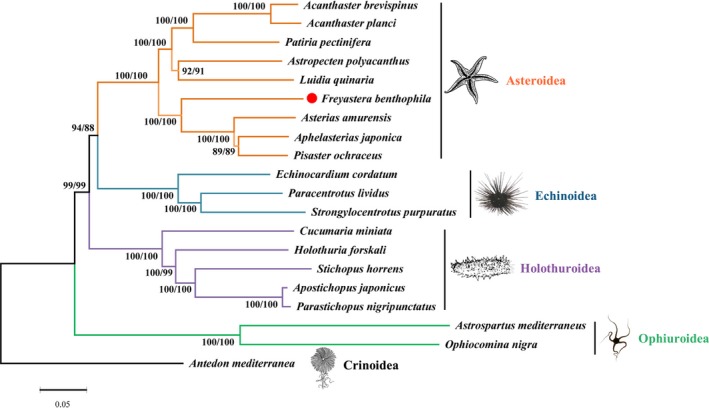
Phylogenetic trees based on the concatenated amino acids of 13 protein‐coding genes. The branch length is determined with NJ analysis. *Antedon mediterranea* was used as out‐group. NJ (left number) and ML (right number) bootstrap values are given for each branch. The red dot highlights the species sequenced in this study

The gene order and transcriptional orientation of the eight Asteroidea species are completely identical to each other, so the mt genome structures would not provide the phylogenetic information. Thus, we performed the phylogenetic analysis using all amino acids of mt protein‐coding genes (Figure 5). Almost all the phylogenetic relationships are supported with high values (NJ/ML bootstraps 99–100). *Acanthaster brevispinus* is first clustered with *A. planci* and then united with *P. pectinifera*; meanwhile, *Astropecten polyacanthus* and *L. quinaria* formed a clade. And these five starfish formed the Valvatacea clade. Then, *A. japonica* is first clustered with *Pisaster ochraceus* and then united with *A. amurensis*. Finally, *F. benthophila* with these three species formed a Forcipulatacea clade. Blake (1987) recognized that Brisingida and Forcipulatida are the two orders within the Forcipulatacea and suggested that they were the most primitive asteroids (Blake, 1987, 1988). Mah and Foltz (2011) described that the largest clade within the Forcipulatacea is formed by the Brisingida and Asteriidae, which forms a clade of deep‐sea and Southern Hemisphere taxa. In the present study, the results supported the deep‐sea Brisingida as a sister taxon to the traditional members of the Asteriidae, and the branch support values are higher than those in previous studies (Glover et al., 2016; Mah & Foltz, 2011). However, the number of Brisingida species with complete mitogenome is still limited, and more mitogenomes and analysis are necessary to determine the phylogenetic relationship among members of Brisingida.

### 3.6 Positive selection analysis

We examined the potential positive selection in Brisingida lineage because of the colonization of deep‐sea environments which may affect the function of mitochondrial genes. The results of selective pressure analyses are shown in Table 5. When the *ω* ratios for the 13 concatenated mitochondrial protein‐coding genes were tested between the deep‐sea *F. benthophila* and other eight shallow sea starfish, we failed to find a significant difference in their *ω* ratios, which may be due to the large bias of sample sizes (*p *>* *0.05) (Table 5). In addition, in the analyses of individual genes, we found five residues with high posterior probabilities in the *atp8* (8 N, 16 I), *nad2* (47 D, 196 V), and *nad5* (599 N), respectively (Table 5). Similar results have been observed in deep‐sea animals, and the authors concluded that it may be related to the adaptation to environment (Sun et al., 2018; Zhang et al., 2017). Under the deep‐sea extreme environment, survival may require a modified and adapted energy metabolism (Sun et al., 2018).

**Table 5:** Selective pressure analyses of the mitochondrial genes of starfish

Trees	Branch model			Model compared	2△lnL	LRT *p*‐value
	Model	lnL	Estimates of parameters			
NJ/ML	Model 1	−77,922.08746		Model 1 versus Model 0	127.22418	0.00000
	Two ratio	−77,985.69322	*ω*0 = 0.06446 *ω*1 = 0.06371	Two ratio versus Model 0	0.01266	0.91027
	Model 0	−77,985.69955	*ω* = 0.06438			

Gene	Branch site model (BSM)							Model compared	2△lnL	LRT *p*‐value	Positive sites
	Model	lnL	Estimates of parameters								
*atp8*	Model A	−1,269.41181	Site class	0	1	2a	2b	Model A versus Model A null	1.82842	0.17632	8 N 0.996**
			Proportion	0.53865	0.21395	0.17707	0.07033				16 I 0.971*
			Background *ω*	0.08240	1.00000	0.08240	1.00000				
			Foreground *ω*	0.08240	1.00000	151.29990	151.29990				
	Model A null	−1,270.32602									
*nad2*	Model A	−7,398.27984	Site class	0	1	2a	2b	Model A versus Model A null	2.54316	0.11077	47 D 0.969*
			Proportion	0.83756	0.10993	0.04641	0.00609				196 V 0.959*
			Background *ω*	0.04253	1.00000	0.04253	1.00000				
			Foreground *ω*	0.04253	1.00000	127.13451	127.13451				
	Model A null	−7,399.55142									
*nad5*	Model A	−12,858.89872	Site class	0	1	2a	2b	Model A versus Model A null	0.34180	0.55879	599 N 0.961*
			Proportion	0.76189	0.18603	0.04185	0.01022				
			Background *ω*	0.04324	1.00000	0.04324	1.00000				
			Foreground *ω*	0.04324	1.00000	1.52724	1.52724				
	Model A null	−12,859.06962									

*Posterior probability >95%; **Posterior probability >99%.

Because ATP synthase directly produces ATP, variation in ATPase protein sequence should influence ATP production (Mishmar et al., 2003; Wallace, 2007). Amino acid variations have been widely reported in the ATPase proteins (da Fonseca et al., 2008; Mishmar et al., 2003; Zhang et al., 2017; Zhou et al., 2014). *Nad2*,* nad4*, and *nad5* are suggested to act as proton‐pumping devices (Brandt, 2006; da Fonseca et al., 2008); thus, mutations in these proteins should influence metabolic efficiency (da Fonseca et al., 2008; Hassanin, Ropiquet, Couloux, & Cruaud, 2009; Zhang et al., 2017). Therefore, we predict that mitochondrial protein‐coding genes, specifically *atp8*,* nad2*, and *nad5*, may play an important role in *F. benthophila*'s adaptation to deep‐sea environment.

## 4 CONCLUSIONS

In this study, we determined the mitogenome of the deep‐sea member *F. benthophila*, which is 16,175 bp in length and encodes 37 genes including 13 PCGs, two rRNA genes, and 22 tRNA genes on the both strands. We described the mitogenome features, codon usage, gene arrangement, phylogenetic analysis, and positive selection of the starfish *F. benthophila*. This study is the first determination of the mitogenome of a deep‐sea member of the order Brisingida and may shed light on the adaptive evolution of Brisingida species to the deep‐sea environment.

## CONFLICT OF INTEREST

The authors declare no conflict of interest.

## AUTHOR CONTRIBUTIONS

Haibin Zhang and Wendan Mu designed the study. Haibin Zhang contributed to the project coordination and collected the samples. Wendan Mu conducted the sequence analyses and drafted the manuscript. Haibin Zhang and Jun Liu helped to draft the manuscript. All authors read and approved the final manuscript.

## DATA ACCESSIBILITY

The complete mitochondrial DNA sequence has been deposited in GenBank (Accession Number: MG563681).
