# Supplementary material for: The first complete mitochondrial genome of the Mariana Trench Freyastera benthophila (Asteroidea: Brisingida: Brisingidae) allows insights into the deep‐sea adaptive evolution of Brisingida
Source: Ecol Evol. 2018 Oct 31;8(22):10673–86. doi: 10.1002/ece3.4427 (PMC6262923; doi:10.1002/ece3.4427)
Supplement: Supplementary file 1 [file ECE3-8-10673-s001.pdf]

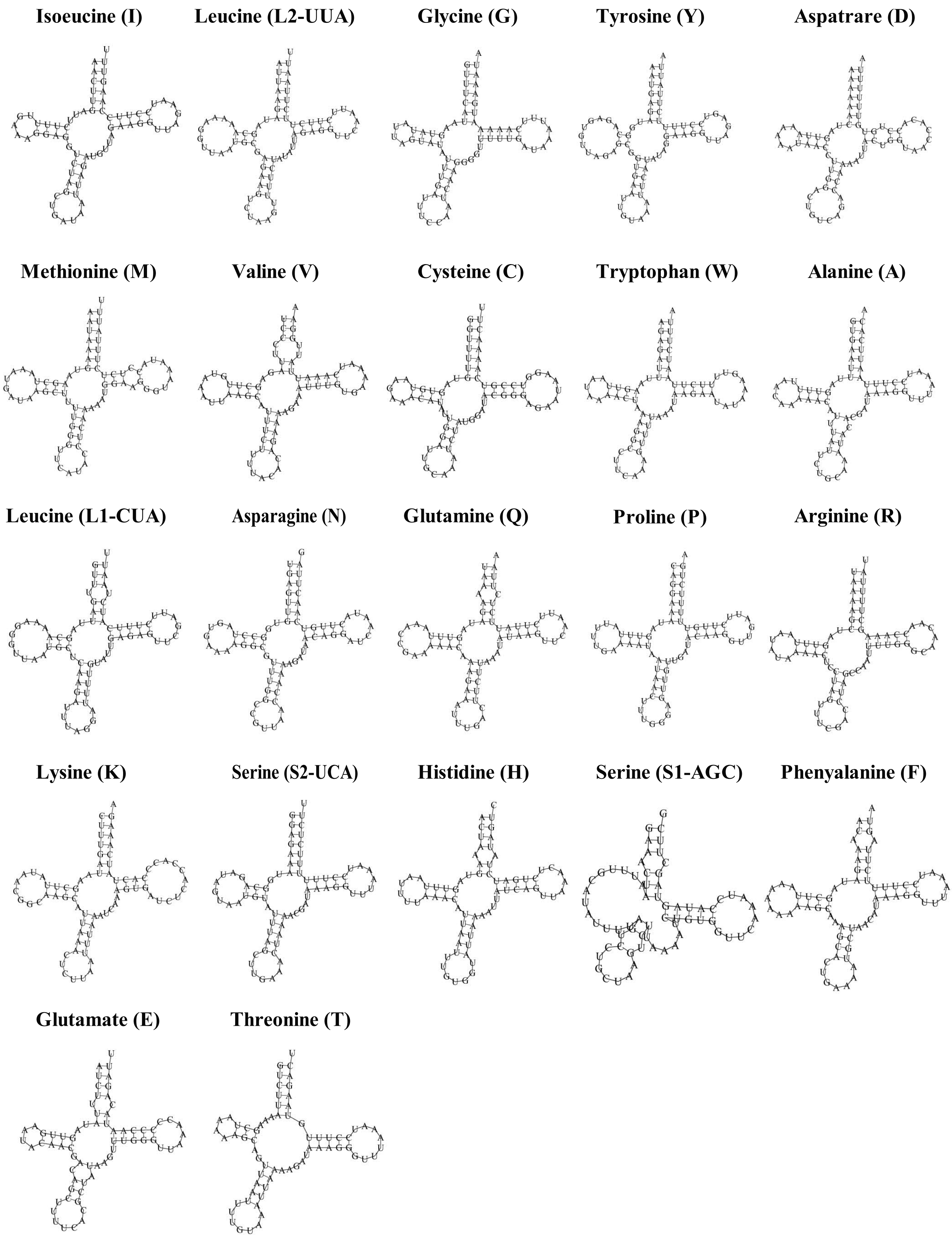
Isoeucine (I)
Leucine (L2-UUA)
Glycine (G)
Tyrosine (Y)
Aspatrare (D)
Methionine (M)
Valine (V)
Cysteine (C)
Tryptophan (W)
Alanine (A)
Leucine (L1-CUA)
Asparagine (N)
Glutamine (Q)
Proline (P)
Arginine (R)
Lysine (K)
Serine (S2-UCA)
Histidine (H)
Serine (S1-AGC)
Phenyalanine (F)
Glutamate (E)
Threonine (T)
